# The Effect of Stress on the Skin Welfare of Lumpfish (*Cyclopterus lumpus* Linnaeus, 1758) Broodstock

**DOI:** 10.3390/ani14213114

**Published:** 2024-10-29

**Authors:** Thor Magne Jonassen, Albert Kjartan Dagbjartarson Imsland, Karin Pittman

**Affiliations:** 1 Akvaplan-niva AS, Oslo Office, Økernveien 94, 0579 Oslo, Norway; tmj@akvaplan.niva.no; 2 Akvaplan-niva AS, Iceland Office, Akralind 6, 201 Kópavogur, Iceland; 3 Department of Biological Sciences, University of Bergen, High Technology Centre, 5020 Bergen, Norway; karin.pittman@uib.no

**Keywords:** reproduction, cortisol, skin health, Q cells

## Abstract

The aim of this study was to build on the work already conducted in optimising lumpfish broodstock temperature, photoperiod and nutrition by providing vital information on the stress relationship between mucosal barrier functions and the sexual development of lumpfish broodstock. From a population of approx. 300 fish of 1587 g (SEM ± 704 g), a sub-population of 20 fish was injected with 30 mg/kg fish cortisol implants on 5 January 2018. The control group was not treated with implants. The stress-induced cortisol implant group showed elevated plasma cortisol over a period of approximately one month. Analyses of mucus cell area and density suggested that induced stress could produce a positive functional response (stimulus) in lumpfish early in the experiment through a gentle increase in the barrier strength of the skin, reduced mucus cell size and increased density of mucous cells, resulting in a strengthening of the respiratory capacity of the gills. Mucous cell density and calculated barrier strength in the skin were significantly negatively correlated with plasma cortisol, while in the gill filaments of females, there was a significant negative correlation between mucous cell density and oestrogen levels. The reduced density of “empty” cells (Q cells) after stress induction indicates that these cells are important for the maintenance of homeostasis (physiological equilibrium). The fish in this experiment were considered more robust compared to previous analyses on smaller lumpfish.

## Simple Summary

The aim of this study was to build on the work already conducted in optimising lumpfish broodstock temperature, photoperiod and nutrition by providing vital information on the stress relationship between mucosal barrier functions and the sexual development of lumpfish broodstock. A group of 20 lumpfish with a mean weight of 1587 g (SEM ± 704 g) were injected with 30 mg/kg fish cortisol implants. The control group was not treated with implants. Analyses of mucus cell density and distribution generally showed that the induced stress produced a positive functional response (stimulus) in lumpfish through a gentle increase in the barrier strength of the skin and reduced mucus cell size and reduced density of mucous cells. The reduced density of “empty” cells (Q cells) after stress induction indicates that these cells are important for the maintenance of homeostasis (physiological equilibrium). This is the first experimental trial that has investigated how induced stress can affect the skin welfare of lumpfish broodstock.

## 1. Introduction

The prerequisite for successful controlled reproduction is controlled broodstock farming, which ensures a good and stable physiological mode in the fish (homeostasis) that provides a predictable response to the control tool. Recently, the lumpfish (*Cyclopterus lumpus* Linnaeus, 1758) has been suggested as a cold-water cleaner fish for the removal of sea lice from Atlantic salmon (*Salmo salar* Linnaeus, 1758). Interest in the use of hatchery-reared lumpfish has increased [1,2,3] rapidly, concurrent with the species being used as a biological delouser for Atlantic salmon. Challenges with disease, wounds and mortality in farmed lumpfish have often been reported [1], indicating cases of poor farming conditions and chronic stress. In addition, there is a large spread in spawning time and fecundity within lumpfish spawning groups [2,3,4,5]. Early in sexual maturity, stress can lead to a reallocation of energy where investment in offspring (gamete production) is given lower priority, but at later stages, stress can lead to the prioritisation of egg production at the expense of somatic growth [6]. Plasma cortisol is one of the most commonly measured indicators of stress and has a direct inhibitory effect on steroidogenesis [7]. Schreck [8] concluded that understanding the influence of stress and the stress response depends on where in the reproductive process the influence occurs and may be relevant for optimising operating routines to ensure good reproductive fitness (reproductive yield, hatching rate and quality of offspring).

The mucosal barriers of skin and gills are the frontline protection in teleosts, containing multitudinous substances for communication with the external world, including microbial and physical impacts. Such mucosal barriers are part of the external immune system and have acted as a communication layer of living cells between aquatic organisms and the environment for over half a billion years [9]. The mucus layer of fish contains a number of immune-relevant compounds, such as lectins, lysozymes and immunoglobulins, which form a biochemical barrier that constitutes the fish’s first line of defence or external barrier against a number of pathogens. Several of these have also been detected in lumpfish [10]. In general, the skin acts like a shield, reflecting the microbiome of surface contacts and impacts, while the gills act as a sentinel guard, comprising 50% of the surface area of the fish, and in constant dialogue with water quality, particles and waterborne microbia [11,12], both can respond to various kinds of stress [13].

Attempts to quantify the factories of skin, gill or gut mucus (the mucous cells) were hampered by descriptive results and traditional counting of the number of cells per variable unit of the measure until the development of quantitative mucosal mapping, which is now trademarked as Veribarr™ (QUNATIDOC, Bergen, Norway), and the verification of barriers [13,14,15,16,17,18]. The use of the number alone as a response descriptor is particularly misleading for biological significance because of the effect of cell size on the functional potential of these cells (Supplementary Table S1). The normal response to chronic, but not acute, stress in the fish skin [14,15,16,17] is reduced volumetric density of mucous cells in the epithelium (MCD, %), increased or exhausted mean area of mucous cells (MCA, μm^2^) and reduced barrier strength and is associated with reduced robustness and welfare. Healthy gills, by contrast, have little need for mucous cells in the respiratory tissue of gill lamellae, whereas the gill filament may secrete somatic substances through its mucous cell population, which is both larger and more abundant than in the lamella [16].

This paper aims to build on the work already conducted in optimising lumpfish broodstock temperature [4], photoperiod [2,3] and nutrition [19] by providing vital information on the stress relationship between mucosal barrier functions and sexual development.

## 2. Materials and Methods

### 2.1. Experimental Fish

Juvenile lumpfish (N = 300, weight range, mean = 8–10 g) produced at Fjord Forsk Sogn, Sogndal, Norway (offspring of wild fish caught in the Sognefjord) were kept at Industrial Laboratory (ILAB) at the University of Bergen until November 2016 and reared under a simulated natural photoperiod for Bergen (60° N) on seawater pumped from 100 m depth outside Nordnes, Bergen and stable temperature (8.5–9.4 °C) and salinity (33.9–34.7‰). Oxygen saturation ranged from 85 to 94%. The fish group was kept in a 3 m tank of 7000 L and fed a commercial marine dry feed (Vitalis Clean 7 broodstock feed: 59% protein, 11.5% fat, 12.5% ash, 0.4% fibre, 1.3% phosphor; Skretting, Fontaine-les-Vervins, France), where pellet size was adjusted according to weight. The fish density was kept lower than 30 kg/m^3^. The fish were raised here for the next 14 months until the start of this trial. The corresponding data on the gonadal development of lumpfish in the present experiment are described in [5].

### 2.2. Stress Induction

At the start of the experiment, 5 January 2018, six undisturbed lumpfish (1587 g, SEM ± 704 g) from a base population of approx. 300 fish contained in a 6000 L tank were sampled for blood, skin tissue and gills. Thereafter, two groups of 20 lumpfish each were randomly collected from the base population. Before moving the two groups to separate tanks (2500 L), they were injected with 30 (“high”) and 15 mg (“low”) cortisol implants per kg fish, and groups were marked with different colours. The cortisol injection was performed in order to test how the introduction of chronically elevated plasma cortisol (stress) affects the natural sexual maturation cycle. The cortisol implant (hydrocortisone, H4001 SIGMA, CAS Number 50-23-7) was diluted to 20 mg cortisol per ml vegetable fat (mixture of vegetable oil and hydrogenated fat in a ratio of 1:1) and injected intraperitoneally [20]. The fluorescent colour mark (Northwest Marine Technology Inc., Anacortes, WA, USA) was injected subcutaneously at the top of the head.

#### 2.2.1. Pilot Trial

The dose–response was tested in a pilot trial between the two injected groups to determine what cortisol dosage to use in the trial. Plasma cortisol measured on 19 January from 6 fish from each group showed that the “high” group had 931.2 (SEM ± 204), while the “low” group had 320.3 (SEM ± 165) ng/mL plasma cortisol. These levels decreased to 95.9 (SEM ± 27.2) and 44.1 (SEM ± 20.4) on 2 February and further decreased to 25.4 (SEM ± 10.1) and 6.7 (SEM ± 2.0) on 15 February for “high” and “low” groups, respectively. The plasma cortisol levels in the two experimental groups were significantly different at all these time points (one-way ANOVA, *p* < 0.05). There was no mortality in any of the groups. Due to the higher and more prolonged stress level in the “high” group, this group was selected for further comparison with unstressed group, after which they were transferred to the base population under common garden conditions on 15 February. The “low” dose group was euthanised at a lethal dose of 500 mg/L of Metacaine (MS222), followed by bleeding.

#### 2.2.2. Experimental Groups

The unstressed base population (reference group) was measured on 5 January, 25 January, 23 February and 22 March (Table 1) as part of a larger sampling scheme investigating the whole sexual maturation cycle of lumpfish [5], and following plasma cortisol (ng/mL) values were measured: 20.16 (SEM ± 9.92), 7.9 (SEM ± 1.9), 11.5 (SEM ± 2.4) and 23.1 (SEM ± 2.7), respectively.

To reduce the number of fish sacrificed and reduce the risk of stress due to handling, stressed and unstressed fish were, with a few exceptions, sampled at different dates (see Table 1). The cortisol measurements from the reference fish were derived from blood sampled for the sex steroid analyses presented in [5]. Their data show that stress was induced during mid to late vitellogenesis (ovarian stage 4 to 5) in the present study.

### 2.3. Sampling Protocol

During sampling, 6 fish from each group (3 fish at a time) were netted from the tank and deposited into a 20 L tub of seawater with 200 mg/L Metacaine added for 2–5 min until the fish lost equilibrium and were apathetic. At each time of sampling, the fish were weighed, lengths were measured, and approximately 1 mL of blood was taken with 2 mL heparinised syringes ventrally from the caudal peduncle just behind the caudal aorta and transferred to Eppendorf tubes. The samples were then centrifuged (13,000 rpm for approx. 5 min), and blood plasma was pipetted into new Eppendorf tubes and stored on liquid nitrogen (−80 °C).

Some of the samples were also analysed for steroids (E2 and 11-ketotestosterone). These are reported together with detailed information on the gonadal maturation process [5] on fish from the present experiment. The schedule for blood samples and other analyses is given in Table 1. Due to the limited number of fish in the experiment, there was a spartan withdrawal of lethal biopsies of skin and gills. Samples were collected for mucosal barrier analyses at three timepoints: 5 January from 6 unstressed reference group fish (U), 15 February from 6 stressed fish (S), and 22 March from 6 fish from both groups, while on the final two dates (23 April and 4 May), 6 fish were sampled from each group for blood and gonad analyses. For each euthanised fish, a dorsal skin biopsy of 1.5 cm × 2.0 cm was taken from the right side between two rows of ossicles, and second gill arch from the right gill was excised. The samples were fixed in 4% phosphate-buffered formalin prior to processing and analysis for mucosal barrier health by the standardised Veribarr™ method [15,17]. The same fish examined for mucosal barrier health were also examined for a set of external visual welfare indicators, according to an assessment index defined in [21]. The parameters examined were deformities or ulcers on suction disc, dorsal fin (comb), caudal fin, pectoral fin, posterior dorsal fin, snout/mouth, head, gill lid, eye (cataract, injuries/bleeding), skin on the left side, and skin on the right side. When compiling and weighing, a score from 1 to 4 for each indicator was given, depending on the extent and severity of each condition (1: good condition; 4: severe condition). The index has proven to be a good practical tool for assessing welfare.

Gonads were excised from 6 females and 6 males on 22 March and 23 April and weighed for gonadosomatic index (GSI), which was calculated as follows:

GSI = gonad weight (g) × 100/total body weight (g)

### 2.4. Analyses

Plasma was diluted at 1:100 and analysed in duplicate. Plasma cortisol (ng/mL) was analysed with the DetectX^®^ Cortisol Enzyme Immunoassay Kit (Arbor Assays, Ann Arbor, MI, USA). The standard curve with a range from 0 to 320 ng/mL was based on a standard with a known cortisol concentration. The detection limit has been tested at 0.045 ng/mL. Samples outside the lower limit of the standard curve were set at <0.05 ng/mL. Steroids (oestrogen (E2) and testosterone (11-KT) were extracted from blood plasma according to method modified by [22].

The formalin-fixed samples of skin and gills were purified in phosphate-buffered saline (PBS) and dehydrated in ethanol (50%, 70% and 80%), followed by paraffin-embedding, tangential sectioning [15] and staining with Periodic Acid Schiff (PAS)–Alcian Blue [14,15]. High-resolution digital scans of one section per tissue per fish were used to determine the mean size of mucous cells (MCA), the volumetric density of mucous cells in the epithelium (MCD), and, from these measures, derive the defence activity or barrier status (DA) of the tissue using the formula DA = (1/(MCA:MCD)) × 1000).

Analysis also revealed another dominant and large cell type in the skin, referred to as Q cells, which can have obvious internal structures [23] or little or no content. These resemble club cells [24] but differ in that they may have internal structures; they do not contain collagen or glycoproteins when staining with Massons trichrome and PAS-AB; and they can change location, size and volumetric density in the epithelium in response to treatment and habitat [23]. These Q cells were measured and registered using the same method as for mucous cells in the skin.

### 2.5. Statistical Analyses

Statistical tests were performed in STATISTICA^TM^ 14.0 (Tibco, 3307 Hillview Avenue, Palo Alto, CA, USA) and Microsoft Excel, version 16.0. To assess normality of distributions, a Kolmogorov–Smirnov test [25] was used, and homogeneity of variances was tested using Levene’s F test [26]. One-way ANOVA [26] was used to investigate the effect of treatment. Significant ANOVA was followed by Student–Newman–Keuls post hoc test (SNK) to determine the differences between the groups. Possible correlation between skin mucosal epithelium cell state, gill lamellae and gill filament with sex hormone status was calculated using the simple correlation coefficient [26], and the correlation coefficient was tested for possible significance with a Student’s *t*-test [26]. A significance level (α) of 0.05 was used if not stated otherwise.

### 2.6. Ethics

This study was conducted according to the Animal Welfare Act (LOV-2009-06-19-97). The present trial was approved by the local responsible laboratory animal science specialist under the surveillance of the Norwegian Animal Research Authority (NARA) and registered by the authority by the following registration numbers: FOTS 23018.

## 3. Results

### 3.1. Stress Development

**Figure 1:**
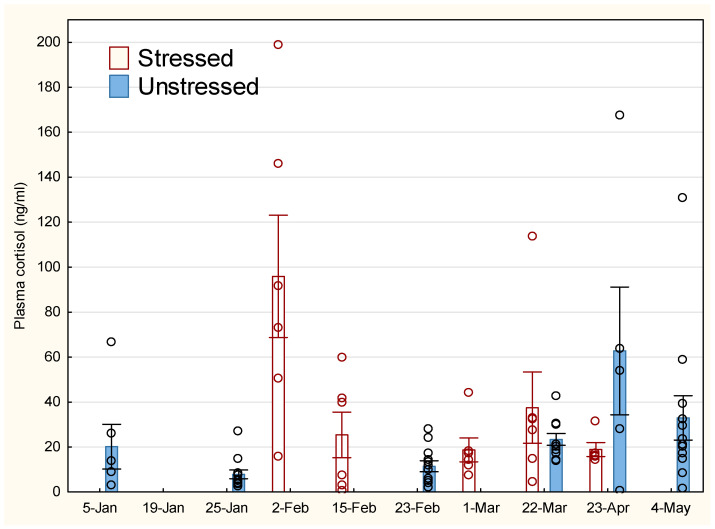
Development in plasma cortisol for stressed and unstressed lumpfish. The vertical line on the bars representing mean values indicates standard error (SEM). Open circle sign indicates measurements of individual fish.

The development in stress response for the unstressed (U) and stressed (S) groups is shown in Figure 1, where S received elevated cortisol up to 15 February compared to U. There was a tendency towards increased cortisol levels in U later in the experiment. There was no significant difference between the groups on 22 March, but a tendency towards lower cortisol levels in S on 23 April.

**Figure 2:**
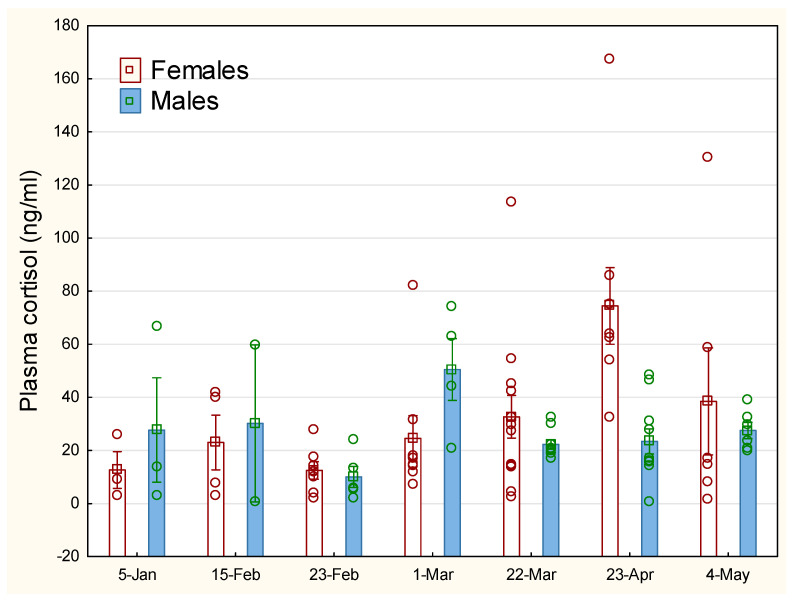
Sex-specific development in plasma cortisol for lumpfish. The vertical line on the bars representing mean values indicates standard error (SEM). Open circle sign indicates measurements of individual fish.

Gender-specific variation in cortisol for those measurement points where reliable information on gender is available shows little variation in stress levels between the sexes for pooled treatment groups (Figure 2), except on 23 April when female fish had significantly (*p* < 0.01) higher cortisol levels (74.5 ng mL^−1^) compared to male fish (24.2 ng mL^−1^).

### 3.2. Sexual Maturation

**Figure 3:**
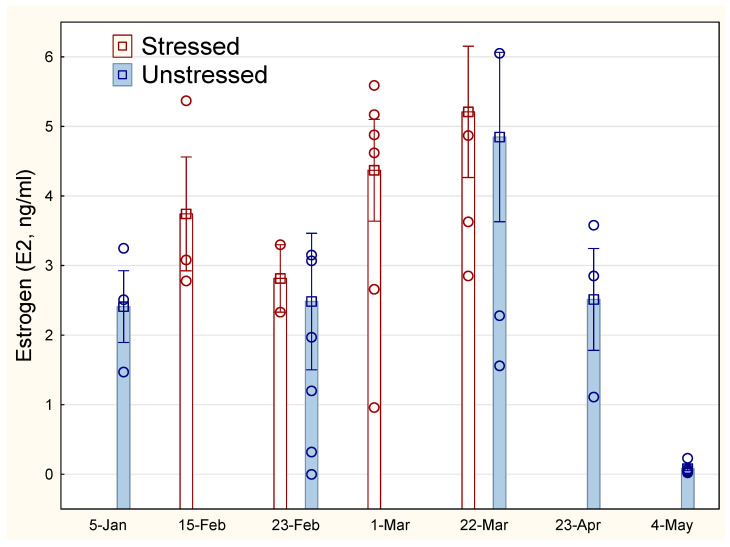
Development in oestrogen (E2) for stressed and unstressed lumpfish. The vertical line on the bars representing mean values indicates SEM. Open circle sign indicates measurements of individual fish.

The sexual maturation development for female fish shown as the concentration of oestrogen (E2) in S and U is shown in Figure 3, with an increase in oestrogen for all groups up to a peak around 22 March. There were no statistical differences in oestrogen between the groups and measurement points from 23 February to 22 March. No weight differences were found between the females of the two experimental groups (one-way ANOVA, *p* > 0.5).

**Figure 4:**
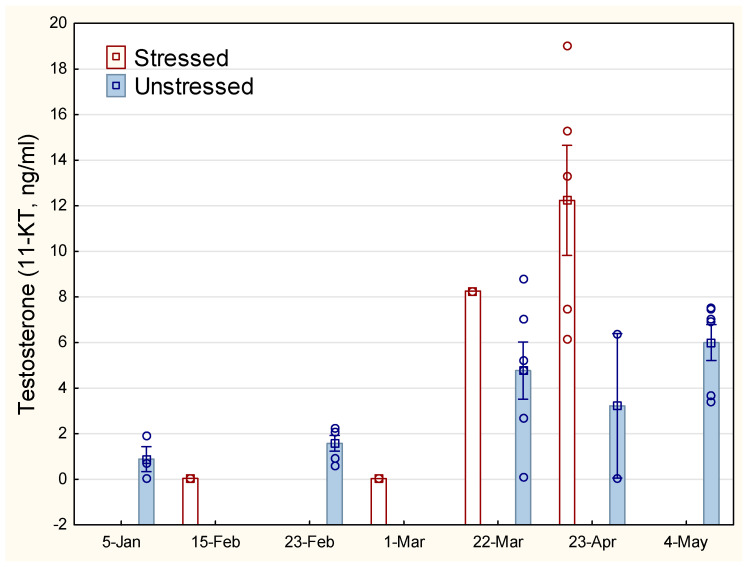
Development in 11-ketotestosterone for stressed and unstressed lumpfish. The vertical line on the bars representing mean values indicates SEM. Open circle sign indicates measurements of individual fish.

The development in 11-ketotestosterone (11-KT) for male fish increased from about March (Figure 4). Large individual variation within each group and a small number of male fish makes the analyses uncertain. Nevertheless, there is a clear tendency towards a higher and more limited peak in testosterone for stressed male fish in March–April compared with unstressed males.

There was no correlation between the hormone status of male fish (11-KT, R^2^ < 0.01, *p* = 0.62, N = 41) or female fish (E2, R^2^ < 0.001, *p* = 0.77, N = 57) and stress levels (plasma cortisol).

There were no significant differences (one-way ANOVA, *p* > 0.55) in mean (±SEM) GSI for lumpfish females in the S-group (14.6% ± 1.4) and the U-group (12.1% ± 3.6) on either 22 March or 23 April (S-group = 16.9% ± 2.2), U-group = 19.0% ± 4.1).

### 3.3. Mucosal Barrier Responses

**Figure 5:**
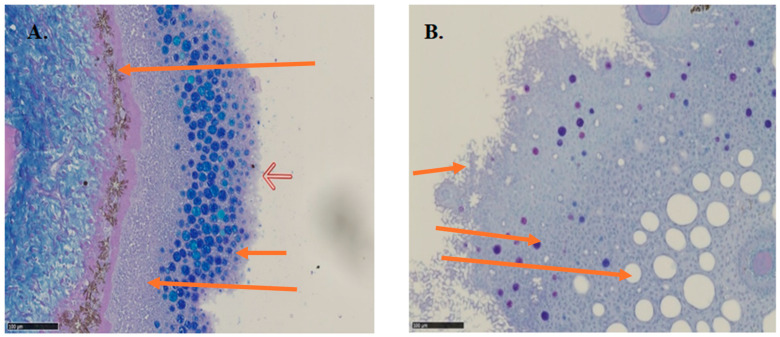
Section of skin sample from lumpfish with slime cells ((**A**): many small cells stained blue) and “Q-cells” ((**B**): “empty” large cells). The sections are stained with Periodic Acid Schiff (PAS)–Alcian Blue. Black scale bar = 100 μm. (**A**) Upper arrow = pigment layer; hollow arrow = petals of special epithelial surface cells; short arrow = mucous cells stained blue; long arrow = epithelial cells. (**B**) Upper arrow = petals of special epithelia surface cells; middle arrow = mucous cells stained blue; bottom arrow = Q cells with no stain inside.

An example of tangentially sectioned lumpfish skin with a “fronded” cell structure on the surface, small mucus cells (small cells, coloured blue, Figure 5A) distributed in the superficial layer of the skin surrounded by large “empty” Q cells (unstained large cells, Figure 5B) is shown in Figure 5. Q cells are found only in the skin, not on gill tissue.

#### 3.3.1. Skin

Skin samples from males and females were combined in order to achieve a sufficient number for analyses (N = 12 for each group, Table 2). There was an impact of stress history on the size of the mucous cells, where stressed fish showed a significantly smaller MCA on 15 February compared with unstressed fish at the beginning of the experiment on 5 January (133 μm^2^ vs. 112 μm^2^, one-way ANOVA, *p* < 0.05). However, there were no significant differences in MCD or barrier strength at these early points, and no differences between stressed and unstressed groups were found on the final date of 22 March (Table 2), when all fish were in oocyte stage 7 (Figure 3).

#### 3.3.2. Gill Lamellae

The gill lamellae of stressed fish showed significantly smaller MCA (67 μm^2^) on 15 February compared to the unstressed group (90 μm^2^) on 5 January (one-way ANOVA, *p* < 0.05, Table 2), but no significant difference in MCD and barrier strength. There were no differences in MCA and MCD between the groups on 22 March. There was a tendency towards somewhat reduced barrier strength over time.

#### 3.3.3. Gill Filaments

On 5 January, the unstressed fish gill filaments had significantly larger cells at greater density and barrier strength than did the stressed group on 15 February (Table 2). For the stressed group, MCA showed a small reduction over time from 47 μm^2^ to 41 μm^2^ (*p* < 0.001), a small increase in MCD from 2.3% to 2.7% and an increase in barrier strength from 0.5 to 0.6. This contrasts with the significant reductions over time in the unstressed group filament cell size (MCA from 89 to 50 μm^2^), in density (MCD from 10.6% to 3.5%) and in barrier strength (from 0.12 to 0.07).

### 3.4. Q Cells

When analysing the skin samples, the Q cell appeared 2–10 times more abundant and 10–20 times larger than the mucous cells and thus visually more dominant (Table 2, Figure 5B). The size (QCA) of the Q cells was significantly larger for stressed fish on 15 February (2136 μm^2^) compared to unstressed fish on 5 January (1677 μm^2^), and there was a significantly elevated QCA for stressed fish on 15 February (2136 μm^2^) and 22 March (2666 μm^2^) compared with unstressed fish (1677 μm^2^) on 5 January (*p* < 0.05).

The density (QCD) of the Q cells was initially higher in unstressed fish (46.2% vs. 16.5% for stressed fish (*p* < 0.05), giving an increased number of small Q cells in the skin mucus of unstressed lumpfish. There was a clear tendency towards reduced QCD over time for unstressed fish, which was significantly lower (23%) on 22 March compared to initial values (46%; *p* < 0.01) and both groups had similar Q-cell densities on 22 March (23% and 22%).

A change in the prevalence of Q cells and mucous cells was observed as a result of stress induction. Before stress, the mucous cells were confined to a thin band in the epidermis, and the Q cells gathered in a deeper surface band, while after stress and over time, the mucous cells were also distributed in the deeper epidermis, closely associated with the large Q cells that had been reduced in abundance.

Correlations between skin mucosal epithelium cell state, gill lamellae and gill filament with sex hormone status (cortisol, oestrogen, and 11-keto-testosterone) are shown in Table 3. There was a significant negative correlation between skin cell density (MCD) and cortisol and between skin barrier status and cortisol. There was a significant (*p* < 0.05) negative correlation between the density of Q cells in male lumpfish skin (QCD) and 11-KT, indicating a possible sex-dependent action of the Q cells. In addition, there was a nearly significant (*p* = 0.06) positive correlation between the size of Q cells (QCA) and plasma cortisol. Thus, the differential action of these two cell types to (chronic) increased cortisol seems to be to reduce mucous cell size and density but to increase the size of the Q cells, as well as to mobilise a repositioning in the skin tissue.

## 4. Discussion

### 4.1. Stress and Mobilisation of Energy Reserves

Comparisons of stress responses between different farmed species [27] have shown a general primary stress response with increased cortisol, but these have also shown that there is a species-specific variation in patterns and degree of responses to stress and stress tolerance. In comparative stress trials with Atlantic salmon, Ballan wrasse (*Labrus bergylta* Ascanius, 1767) and lumpfish, lumpfish showed the lowest response to crowding stress (about 200 ng/mL cortisol), where salmon and Ballan wrasse had a maximum of 800–1000 and 500–700 ng/mL, respectively [28]. Compared with species such as salmon and Ballan wrasse, with a clear flight response to threats and where cortisol contributes to the rapid mobilisation of energy stores, lumpfish have a more sedated lifestyle and another evolutionary defence strategy against threats, which does not require the mobilisation of energy reserves [29].

Reproduction is encompassed by some of the most energy-intensive processes, and great flexibility in terms of energy allocation gives the fish the opportunity to set priorities in relation to maintenance metabolism, immune defences, somatic growth and gonad development [30]. Chronic stress can, therefore, influence the fish’s sexual maturation strategy through its influence on the maintenance metabolism of the broodstock and the nutritional status of the oocyte and embryo [30,31]. If stress in lumpfish causes little mobilisation of energy stores, it is conceivable that stress has less impact on the course of sexual maturation compared to species where stress causes high energy mobilisation. This is consistent with the overall development of sex hormones in this trial.

### 4.2. Influence of Stress Induction on Maturation

While there is ambiguity in the spawning window of lumpfish within the North Atlantic [4,32,33], the population of lumpfish in this study began to ovulate in March–April. A pattern of gradual increase in cortisol towards a peak around the time of ovulation in March–April for lumpfish [5]. Such a development shows that cortisol is part of the natural endocrine control of reproduction and can be produced in female and male gonads [34]. This is consistent with the biological effects of cortisol on mechanisms prior to ovulation, including changes in metabolic parameters, such as increased hepatic amino acid catabolism and increased release of glucose [34]. For lumpfish in this study, the higher levels of plasma cortisol for female fish before spawning (ovulation) at the end of April ([5] present study) suggest that this metabolic regulation is more demanding for female fish than for male fish.

The absence of correlations between the levels of plasma cortisol and the two sex hormones (E2 and 11-KT) in the present study supports the observations that increased stress did not affect sexual maturation in lumpfish late in their sexual maturation cycle. This may possibly be related to the lower energy mobilisation of stressed lumpfish and, thus, a lower vulnerability in relation to ensuring energy allocation for egg growth and sperm production under increased stress. Another possible explanation is that the effects of cortisol on sexual maturation are primarily associated with the early stages of the sexual maturation cycle and not with the maturation stage itself [7].

### 4.3. Skin Health

Mucus-producing cells, called mucous cells or goblet cells, in barrier tissues of the skin, gills and intestinal tracts can vary in abundance and size depending on the environment, tissue, gender, life stage and physiological state [35]. The sampling site for lumpfish skin samples based on the QuantiDoc method has been standardised. In this experiment with lumpfish, the observed reduction in mucus cell size in the skin after stress inducement is, therefore, an expected functional response to stress in healthy fish—small cells can be efficiently generated and can “wash off” surface irritants through rapid turnover [36]. These results of reduced skin protection after stress somewhat parallel those of wild shorthorn sculpins exposed to environmental heavy metals, where skin barrier status was reduced with increasing levels of background lead and zinc, while in the gill filaments, the mucous cell significantly increased with increasing toxin levels and was positively correlated with liver lead levels [16]. When a stimulus overwhelms the constructive response, the skin (or “shield”) may either reduce mucous cell production in favour of literal shielding or increase the mucous cell size and abundance to increase the production of immune substances but simultaneously weaken the biotensegrity [37] of the skin barrier.

### 4.4. Gill Health

In general, the gills of lumpfish responded to chronic cortisol (stress over time) by decreasing lamellar mucous cell size, density and barrier strength, acting unlike shorthorn sculpin gills and thus suggesting a lumpfish-specific response [16]. There was also a tendency towards a negative correlation between barrier status and chronic cortisol in the gill filament, supporting the role of filamental mucous cells as a site of secretion of somatic substances through perhaps rapid turnover of increasingly smaller cells [16].

In comparison with the data on large broodstock of lumpfish in this experiment, previous studies of smaller lumpfish, between 36 and 103 g [38], have shown smaller and fewer mucus cells in the skin (43–98 μm^2^, 0.7–13.1%). However, in the gill filaments of small lumpfish, the mucous cells were of the same order of magnitude or larger than in this experiment (93–111 μm^2^) and somewhat higher density (11.8–13.5%). The gill lamellae of small lumpfish also had mucus cells that were larger than in this experiment (89–105 μm^2^) but similar or somewhat higher density (4.3–7.6%). This indicates that the broodstock in this experiment had potentially better welfare compared to previously analysed groups of small lumpfish and that lumpfish also respond to habitat conditions [23]. In the present experiment, no size-related differences in skin mucus cell status were found.

It is conceivable that mucus cell status generally reflects good fish welfare based on good farming conditions in this experiment, regardless of treatment, with little handling stress and solid ground to rest on. Experiments with lumpfish in cages showed that lumpfish that did not receive a substrate to attach to (hide) had larger skin mucous cells and lower cell density compared to lumpfish that were given a sitting substrate [23].

### 4.5. Q Cells

The Q cells observed only in the skin were 10–15 times larger and had 3–10 times higher density than the mucous cells. This apparent dominance over the smaller mucus cells with more limited prevalence indicates a prominent role for the Q cells in the regulation of homeostasis and in the innate immune response. The change in the physical location of these Q cells in response to stress indicates a regulated mechanism of cell propagation and an involvement in communication between cells at the tissue level. Ytteborg et al. [24] found that in immature lumpfish, mucous and club (Q) cells exposed to hydrogen peroxide changed their relative positions, and Q cells appeared more in the superficial layers of the skin. The Q cells in the present study were smaller (1295–1682 μm^2^) than previously observed on lumpfish between 36 and 103 g [38] but with a similar density of cells (16–40%). In the study of Jonassen et al. [38], groups of smaller lumpfish were transferred from nursery to sea cages, and there was no clear association with stress (plasma cortisol). For the Q cells, there was a systematic change after transfer to cages [38], and it was concluded that the development in Q cells in lumpfish may be a useful response variable in relation to the environment but that it is very context-specific, such that, e.g., the background of the fish (environment, genetics, nutrition, etc.) is likely to influence the environmental response.

These Q cells have also previously been referred to as vacuoles [39] or club cells and have been shown to vary in abundance, size and distribution between individuals, where the typical feature was numerous vacuoles layered in the epidermis for small fry (<5 g) and fewer, smaller and more dispersed vacuoles in larger fish (>50 g). Whether the observed variation in Q cells was associated with stress and varying environmental conditions was not investigated in the study of Ytteborg et al. [24]. Overall, these previous observations on juvenile lumpfish (>50 g) are consistent with the picture from broodstock in the present study. The analyses of mucus cell status indicate that induced stress produced a positive functional response (stimulus) in lumpfish through a gentle increase in the barrier strength of the skin and reduced mucus cell size and increased density of mucus cells, resulting in a strengthening of the respiratory capacity of the gill vanes. The reduced density of “empty” cells (Q cells) after stress induction indicates that these cells may have an impact on the maintenance of homeostasis, but there is a need for further study of both the function and content of these cells.

## 5. Conclusions

The stress-induced cortisol implant group showed elevated plasma cortisol over a period of approximately one month, which coincided with early and middle vitellogenesis. This did not affect the sexual steroid development of the lumpfish. Mucous cell density and calculated barrier strength in the skin were significantly negatively correlated with plasma cortisol, while in the gill filaments of females, there was a significant negative correlation between mucous cell density and oestrogen levels. The density of skin Q cells in males was significantly negatively correlated with 11-Ketotestosterone levels. Analyses of mucus cell density and distribution generally showed that the induced stress produced a positive functional response (stimulus) in lumpfish through a gentle increase in the barrier strength of the skin and reduced mucus cell size and reduced density of mucous cells. The reduced density of “empty” cells (Q cells) after stress induction indicates that these cells are important for the maintenance of homeostasis (physiological equilibrium). We recommend that standards for skin and gill health (mucosal barriers) be applied to all fish in order to establish baseline levels of protection in any farming or natural system.

## Figures and Tables

**Table 1:** Sampling protocol for blood parameters, skin and gill mucus cells, welfare scores, cataracts and GSI (gonadosomatic index) for the unstressed (U) and stressed (S) groups.

Sampling Date	5 Jan		19 Jan		25 Jan		2 Feb		15 Feb		23 Feb		1 Mar		22 Mar		23 Apr		4 May	
Group	U	S	U	S	U	S	U	S	U	S	U	S	U	S	U	S	U	S	U	S
Sampling type																				
Blood	X			X	X			X	X	X	X			X	X	X	X	X	X	
GSI	X									X					X	X	X	X		
Stripping																			X	X
Skin and gill	X									X					X	X				
Welfare score	X									X					X	X				
Analyses																				
Plasma cortisol	X			X	X			X		X	X			X	X	X	X	X	X	
Plasma steroids	X									X	X			X	X	X	X	X	X	

**Table 2:** Overview of mean values (±SEM) from the mucus cell analyses from skin, gill lamellae and gill filaments from stressed and unstressed groups of lumpfish brood-stock. N = 12 for all groups.

	5 January	15 February	22 March	
Skin	Unstressed	Stressed	Unstressed	Stressed
MCA	133 (±8)	112 (±14)	126 (±14)	121 (±21)
MCD	7.7 (±2.8)	8.6 (±1.6)	5.9 (±2.7)	5.9 (±2.8)
Barrier strength	0.057 (±0.019)	0.078 (±0.014)	0.047 (±0.019)	0.045 (±0.012)
QCA, Q cells	1611 (±324)	2136 (±510)	2452 (±458)	2666 (±482)
QCD, Q cells	46 (±9)	17 (±11)	23 (±17)	22 (±4)
Gill lamellae				
MCA	90 (±12)	67 (±16)	59 (±3)	59 (±12)
MCD	10.4 (±2)	7.7 (±2.8)	5.7 (±1.7)	5.2 (±3.1)
Barrier strength	0.12 (±0.04)	0.11 (±0.02)	0.09 (±0.02)	0.08 (±0.03)
Gill filaments				
MCA	89 (±15)	47 (±9)	50 (±7)	41 (±7)
MCD	10.6 (±4.6)	2.3 (±2)	3.5 (±1.2)	2.7 (±1.3)
Barrier strength	0.12 (±0.05)	0.05 (±0.01)	0.07 (±0.02)	0.06 (±0.02)

Abbreviations: MCA, size of mucous cells in epithelium; MCD, mean volumetric density of mucous cells in the epithelium; QCA, size of Q cells; QCD, density of Q cells.

**Table 3:** Relationship (correlations) between cell status in mucous epithelium (first line defence) hormone status (cortisol, oestrogen (E2) and 11-keto-testosterone (11-KT)).

Analyses, Types	Plasma Cortisol (N = 29)			11-KT, Males (N = 41)			E2, Females (N = 57)		
Skin	b	R^2^	*p*	b	R^2^	*p*	b	R^2^	*p*
MCA	−0.14	0.02	0.49	−0.02	0.00	0.67	−0.20	0.04	0.43
MCD	−0.44	0.19	*<0.05*	−0.27	0.07	0.08	−0.30	0.09	0.23
Barrier strength	−0.45	0.20	*<0.05*	−0.29	0.08	0.08	−0.33	0.11	0.20
Areal Q cells	0.35	0.12	*0.06*	0.38	0.14	0.75	0.33	0.11	0.20
Density Q cells	0.10	0.01	0.61	−0.59	0.35	*<0.05*	−0.39	0.16	0.13
Gill lamellae									
ECA	−0.24	0.06	0.22	−0.48	0.23	0.12	−0.22	0.05	0.39
ECD	−0.25	0.06	0.20	−0.32	0.10	0.31	−0.16	0.03	0.53
Barrier strength	−0.20	0.04	0.31	−0.13	0.02	0.69	−0.02	0.00	0.93
Gill filaments									
ECA	−0.10	0.01	0.60	−0.55	0.30	0.06	−0.43	0.19	0.08
ECD	−0.31	0.09	0.11	−0.42	0.17	0.18	−0.47	0.23	0.05
Barrier strength	−0.36	0.13	*0.06*	−0.06	0.00	0.86	−0.39	0.15	0.12

Abbreviations: MCA, size of mucous cells in epithelium; MCD, mean volumetric density of mucous cells in the epithelium; ECA, cell size in gill lamellae and gill filaments; ECD, % density of cells in gill lamellae and gill filaments; b, slope coefficient; R^2^, coefficient of determination; *p*, *p*-value. Significant *p*-values as displayed in italics.

## Data Availability

Data can be obtained by contacting the authors.

## Supplementary Materials

The following supporting information can be downloaded at: https://www.mdpi.com/article/10.3390/ani14213114/s1. Supplementary Table S1. The relationship between cell size, cell surface area, cell volume, number of cells and the resulting cell volume. The Table shows that that cell size and the volumetric density of a size of cell in the tissue are more important than the number of cells. Reporting only the number of cells can be misleading for biological functions, by ignoring the resultant surface area for transmembrane mucins and membrane receptors and the resultant volume of the cytosol for producing mucins and other proteins. Cell size and volumetric density in the tissue better describe the functioning of that tissue. Typical gill lamellae have mucous cells between 20–60 µ^2^, whereas typical skin mucous cells are from 150–300 µ^2^ and intestinal mucosa present intermediary cell sizes. Five cells of 300 µ^2^ have 15 times more surface area and 58 times more cytosol volume than 5 cells of 20 µ^2^.
