# Supplementary material for: The Effect of Stress on the Skin Welfare of Lumpfish (Cyclopterus lumpus Linnaeus, 1758) Broodstock
Source: Animals (Basel). 2024 Oct 29;14(21):3114. doi: 10.3390/ani14213114 (PMC11545186; doi:10.3390/ani14213114)
Supplement: Supplementary file 1 [file animals-14-03114-s001.zip › animals-3246895-supplementary.pdf]

Supplementary Table S1. The relationship between cell size, cell surface area, cell volume, number of cells and the resulting cell volume. The Table shows that that cell size and the volumetric density of a size of cell in the tissue are more important than the number of cells. Reporting only the number of cells can be misleading for biological functions, by ignoring the resultant surface area for transmembrane mucins and membrane receptors and the resultant volume of the cytosol for producing mucins and other proteins. Cell size and volumetric density in the tissue better describe the functioning of that tissue. Typical gill lamellae have mucous cells between 20-60 μ^2, whereas typical skin mucous cells are from 150-300 μ^2 and intestinal mucosa present intermediary cell sizes. Five cells of 300 μ^2 have 15 times more surface area and 58 times more cytosol volume than 5 cells of 20 μ^2.

Area (μ^2)	Radius (μ)	Surface area sphere (μ^2)	Volume sphere (μ^3)	Number of cells	Surface area in unit (μ^2)	Volume in unit (μ^3)
20	2.5231	80	67.2835	5	400	336.4176
		80	67.2835	10	800	672.8353
50	3.9894	200	265.9615	5	1000	1329.8076
		200	265.9615	10	2000	2659.6152
100	5.6418	400	752.2527	5	2000	3761.2638
		400	752.2527	10	4000	7522.5277
150	6.9098	600	1381.9766	5	3000	6909.8830
		600	1381.9766	10	6000	13819.7660
200	7.9788	800	2127.6921	5	4000	10638.4608
		800	2127.6921	10	8000	21276.9216
250	8.9206	1000	2973.5401	5	5000	14867.70095
		1000	2973.5402	10	10000	29735.4019
300	9.7720	1200	3908.8201	5	6000	19544.1005
		1200	3908.8201	10	12000	39088.2010
